# Classification of videocapsule endoscopy image patterns: comparative analysis between patients with celiac disease and normal individuals

**DOI:** 10.1186/1475-925X-9-44

**Published:** 2010-09-04

**Authors:** Edward J Ciaccio, Christina A Tennyson, Govind Bhagat, Suzanne K Lewis, Peter HR Green

**Affiliations:** 1 Department of Medicine, Columbia University Medical Center, 180 Fort Washington Avenue, New York, NY 10032, USA; 2 Department of Pathology, Columbia University Medical Center, 180 Fort Washington Avenue, New York, NY 10032, USA

## Abstract

**Background:**

Quantitative disease markers were developed to assess videocapsule images acquired from celiac disease patients with villous atrophy, and from control patients.

**Method:**

Capsule endoscopy videoclip images (576 × 576 pixels) were acquired at 2/second frame rate (11 celiacs, 10 controls) at regions: 1. bulb, 2. duodenum, 3. jejunum, 4. ileum and 5. distal ileum. Each of 200 images per videoclip (= 100s) were subdivided into 10 × 10 pixel subimages for which mean grayscale brightness level and its standard deviation (texture) were calculated. Pooled subimage values were grouped into low, intermediate, and high texture bands, and mean brightness, texture, and number of subimages in each band (nine features in all) were used for quantifying regions 1-5, and to determine the three best features for threshold and incremental learning classification. Classifiers were developed using 6 celiac and 5 control patients' data as exemplars, and tested on 5 celiacs and 5 controls.

**Results:**

Pooled from all regions, the threshold classifier had 80% sensitivity and 96% specificity and the incremental classifier had 88% sensitivity and 80% specificity for predicting celiac versus control videoclips in the test set. Trends of increasing texture from regions 1 to 5 occurred in the low and high texture bands in celiacs, and the number of subimages in the low texture band diminished (r^2 ^> 0.5). No trends occurred in controls.

**Conclusions:**

Celiac videocapsule images have textural properties that vary linearly along the small intestine. Quantitative markers can assist in screening for celiac disease and localize extent and degree of pathology throughout the small intestine.

## Background

Celiac disease is common, occurring in about 1% of the population worldwide [1]. It is typically diagnosed from assessment of duodenal biopsies obtained at endoscopy after results of serological testing are obtained [2]. The use of serological testing and endoscopic biopsy is expensive and the economics of the diagnosis of celiac disease requires consideration especially as it is being increasingly recognized in developing countries [3]. Videocapsule endoscopy can potentially be used to examine the entire small bowel in detail, allowing for the visualization of mucosal villous architecture. Thus, it has the potential to enable visual assessment of villous atrophy, an important indicator of celiac disease, so that the extent and severity of any mucosal changes in patients with suspected celiac disease can be measured at a resolution that was previously not possible [4,5].

Early work suggests that videocapsule endoscopy can be helpful in the diagnosis of celiac disease [5]. In a series of consecutive patients undergoing videocapsule endoscopy for signs or symptoms suggestive of the disease, classification using a clinical score was described. In this study, duodenal biopsies were first classified according to the modified Marsh's criterion, and capsule findings were evaluated for presence of visual, endoscopic signs compatible with celiac disease (scalloping of duodenal folds, fissures, flat mucosa, and mosaic appearance). Using the duodenal biopsy assessments as the gold standard, the performance characteristics of capsule endoscopy for diagnosis of celiac disease was found to have a sensitivity of 87.5% and specificity of 90.9% [5]. Thus visual scoring of videocapsule images was found to correlate with the gold standard, biopsy. Yet it is still a subjective process that depends on the expertise of the capsule reader. Moreover, clinical scoring is time consuming and laborious. There have been few studies that have attempted to use quantitative image manipulation, as opposed to visual interpretation of videocapsule images [6]. To address this issue, in this study we describe novel markers to gauge differences in small intestinal images in a quantitative manner, for distinguishing celiac patients with small bowel villous atrophy from control patients.

## Methods

### Clinical characteristics and procedure

Retrospective videocapsule endoscopy data was obtained from 11 celiac patients on a regular diet or within 3 months of starting a gluten-free diet. In these patients the diagnostic biopsy, taken while on a regular diet, showed Marsh grade II-IIIC lesions, except in one patient with hemophilia who did not undergo endoscopy and biopsy. Retrospective videocapsule endoscopy data was also obtained from 10 control patients. All patients were evaluated at Columbia University Medical Center, New York, from May 1, 2008 to July 31, 2009. Informed consent was obtained from all patients prior to videocapsule endoscopy. Indications for this procedure include suspected celiac disease, suspected Crohn's disease, obscure bleeding, iron deficient anemia, and chronic diarrhea. Patients under 18 years of age, pregnant women, and those with a history of intestinal obstruction, presence of a pacemaker, or chronic use of non-steroidal anti-inflammatory drugs (NSAIDs) were excluded. Only complete videocapsule endoscopy studies, reaching the colon, were used for analysis. The retrospective data used for quantitative analysis was comprised of a celiac group consisting of 6 female and 5 male patients (mean ages 50.5 and 44.0 years, respectively), and a control group consisting of 6 female and 4 male patients (mean ages 50.0 and 51.5 years, respectively). Retrospective analysis of videocapsule endoscopy data was approved by the Internal Review Board at Columbia University Medical Center.

The PillCamSB2 videocapsule (Given Imaging, Yoqneam, Israel) [7] was used to obtain the small bowel images in the study groups. The system consists of a recorder unit, real-time viewer, battery pack, antenna lead set, recorder unit harness, battery charger, recorder unit cradle, and real-time viewer cable. The capsule is 26 × 11 millimeters in size and the frame rate is set to acquire two digital images per second. All subjects swallowed the PillCam SB2 videocapsule with radio transmitter after a 12 hour fast and wore a small portable recording device. The recorder received radioed images via a sensor array that was transmitted by the videocapsule as it passed through the GI tract. The procedure began in early morning by swallowing the capsule with approximately 200 cc of water, and investigation was terminated either upon arrival of the capsule in the cecum, or after 8 hours. The capsule reached the cecum in all participants. Subjects were allowed to drink water 2 hours after ingesting the capsule, and to eat a light meal after 4 hours. Videos were reviewed and interpreted by an experienced gastroenterologist using an HIPAA-compliant PC-based workstation equipped with Given Imaging analysis software that was also used to export videos for further analysis. Videoclips of 200 frames each acquired from five locations in the small intestine of each patient by the patients' physicians were analyzed retrospectively. The five regions were: 1. duodenal bulb, 2. distal duodenum, 3. jejunum, 4. proximal ileum and 5. distal ileum. The recorded digital information was downloaded to the computer console, and videos from the stomach and the small and large bowel were reviewed using proprietary software.

### Quantitative Analysis

Videoclips created from the patient data were transferred without patient identifiers to a dedicated PC-type computer for quantitative analysis. From each RGB color videoclip, grayscale images (256 brightness levels, 0 = black, 255 = white) with an image resolution of 576 × 576 pixels, were extracted using Matlab Ver. 7.7, 2008 (Mathworks, Natick MA). Both celiac and control patients were found to have some opaque extraluminal fluid and air bubbles in portions of the image frames. Videoclips having opaque extraluminal fluid or air bubbles comprising >10% of the area in the image frames were excluded from development of classifiers to distinguish the celiac videoclips from control videoclips (see below). When lesser amounts of opaque extraluminal fluid or air bubbles, which have the capacity to magnify the surface texture, were present, they were considered as a first approximation to act as random noise for purposes of analysis.

**Figure 1:**
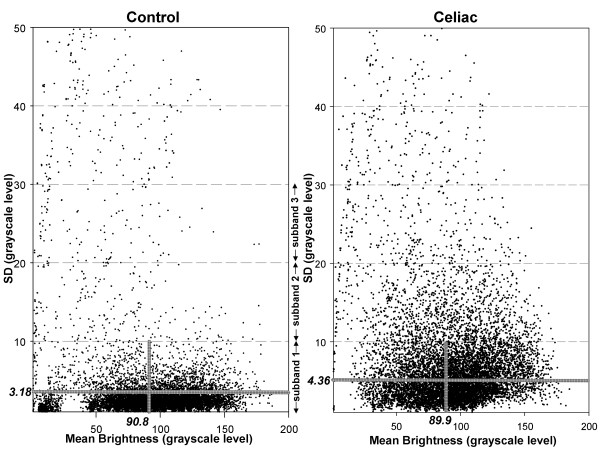
**Plot of surface variability (standard deviation) versus mean surface brightness (units are grayscale level from 1 - 256) from 10 × 10 pixel subimages in one control (left) and one celiac (right) videoclip image frame**. Ranges of 10 grayscale units in standard deviation of pixel brightness are called bands. Many of the subimage values are clustered in band 1 (below 10 grayscale level in standard deviation). However, many celiac subimage values are scattered at higher bands, meaning that there is more variation (higher standard deviation) in the celiac subimages.

The grayscale images were then processed as follows. Subimages of 10 × 10 pixel dimension were extracted from each black-and-white image using computer software developed in-house (56 × 56 subimages were extracted per image, with edges excluded). Mean grayscale brightness level of each subimage and variation in brightness (texture) were calculated for all subimages in the videoclip (56 × 56 × 200 = 627200). For simplicity, subimage texture was defined as the standard deviation in brightness level. An example of a scatterplot of these parameters is shown in Figure 1, where mean grayscale level of each subimage is plotted versus the standard deviation from the mean. For clarity, only a fraction of all subimage values are plotted in the two graphs. The 0-10, 10-20, and 20-30 grayscale level bands in standard deviation (considered to have a low, intermediate, and high degree of image texture, respectively) were used for further analysis, since they contained most of the subimage values and appeared to have morphologically distinct distributions of scatterpoints in celiacs versus controls (Figure 1). The mean and standard deviation in brightness was computed for all bands per image in celiacs and controls. In Figure 1, this is shown for band 1 as denoted by the horizontal and vertical gray lines shown in the scatterplots. The total number of subimages contained in each of the three bands was also tabulated. Thus nine image features in all were used for quantitation, three features for each of three bands.

**Figure 2:**
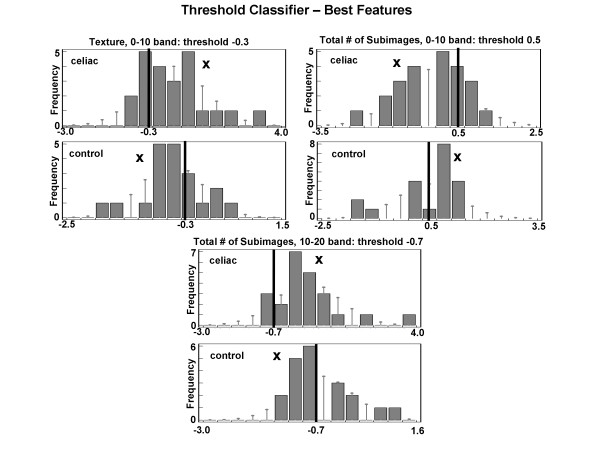
**Development of the threshold classifier to distinguish celiac from control videoclips using exemplar data**. The threshold level T (vertical black line) was determined so as to maximize the sum of the celiac and control videoclips correctly classified (noted by the x). For example in the bottom panel, the threshold is set at -0.7 normalized grayscale units. This threshold level results in the correct classification of all but 3 celiac videoclips (to left of line in upper histogram) and all but 7 control videoclips (to right of line in lower histogram). Of the nine features that were measured (see Methods) the three features shown provided the best threshold classification.

**Figure 3:**
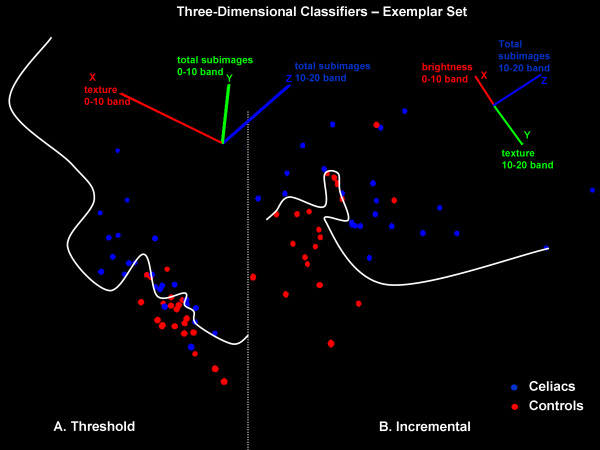
**A. Use of the three-dimensional threshold classifier on a set of exemplar videoclips, with best projection and best nonlinear discriminant function for classification shown**. The features used for classification and orientation of the coordinate axes are noted at top. Most of the control videoclip scatterpoints (red) reside at lower right i.e. approximately aligned with the x-axis, with low degree of texture in the 0-10 band. The nonlinear discriminant function (white curved line) mostly separates the two groups correctly. Only 1 control scatterpoint (red) is clustered with the celiac scatterpoints (blue), and only 2 celiac scatterpoints are clustered with the control scatterpoints (panel A). B. Use of the incremental learning classifier on a set of exemplar videoclips, with best projection and best nonlinear discriminant function for classification shown. The features used for classification and orientation of the coordinate axes are again noted at top.

To develop classifiers, videoclips from the 5 small intestinal locations in 6 celiac and 5 control patients were used as exemplars. Of the 30 celiac videoclips and 25 control videoclips, 23 of the celiacs and 20 of the controls were actually used for analysis. The other videoclips were excluded due to opaque extraluminal fluid or air bubbles >10% of the total pixular area of the images. The mean feature values from each videoclip were pooled from all small intestinal regions and all patients (thus 23 celiacs and 20 controls), and normalized to mean zero and the same range. The histogram of each feature for all celiacs and for all controls was plotted. The threshold level which maximized the number of celiacs on one side versus controls on the other was determined. The top three features with threshold level that maximized the sum of celiacs and controls classified correctly (i.e. maximized accuracy) were incorporated into the classifier. These features are shown in Figure 2. In each panel is a histogram, with frequency of occurrence on the ordinate axis and normalized grayscale level on the abscissa. The histograms show the occurrence of videoclip features, with features from 23 videoclips shown in the celiac histograms and features from 20 videoclips shown in the control histograms. The optimal threshold levels for each histogram are shown as vertical black lines. Using these levels, for the texture 0-10 band (top left panel), 16/23 celiac videoclips and 13/20 control videoclips were correctly classified (accuracy 67.4%). For the total number of subimages in 0-10 band feature, 18/23 celiac videoclips and 13/20 control videoclips were correctly classified - including some within the bar under the threshold line (accuracy 72.1%). For the total number of subimages in the 10-20 band feature, 20/23 celiac and 13/20 control videoclips were correctly classified (accuracy 76.7%). These three best features determined by thresholding were used to construct a three-dimensional feature space in which the feature values for all exemplar videoclips were plotted (23 celiacs and 20 controls, Figure 3A). The space was graphed using map3 d, an interactive scientific visualization tool for bioengineering data (Scientific Computing and Imaging Institute, University of Utah) [8]. It was manually rotated on a computerized grid until the two-dimensional projection which best separated celiac from control points was obtained, as shown. A nonlinear discriminant function was then drawn manually through the points to best separate celiacs from controls (white curved line). Using this exemplar data, the sensitivity was 91% and the specificity was 95% for classification of celiacs versus controls.

A second three-feature classifier was then developed using an incremental learning procedure. A set of three features selected at random (none the same) was initially used for classification. The best two-dimensional projection for separating celiac from normal videoclips with this three feature set was determined using the best possible nonlinear discriminant function as the separation boundary. The first feature in the set was then removed and replaced by each of the remaining six features. The feature which resulted in the best accuracy for classification was kept. The process was then repeated for the second and then the third feature in the set. Although this method can be suboptimal as compared with an exhaustive search, it required testing only 7 + 6 + 6 = 19 combinations (no feature repetition and feature order not important) rather than: n!/[r! (n - r)!] = 84

combinations that would be needed for exhaustive search (r = 3 features chosen from n = 9 features). The best three feature set using this incremental learning method with the exemplars is shown in Figure 3B, and it resulted in a sensitivity of 100% and specificity of 90% for classification of celiac videoclips versus controls. Only one feature (total subimages 10-20 band) is the same for the threshold and incremental learning classifiers (Figure 3).

The threshold and incremental learning classifiers were then used on test videoclip images from 5 celiacs and 5 controls. The same feature sets, feature space rotation, and nonlinear boundaries determined using exemplars were retained for classification of the test videoclips. However, as a check of robustness of the method, clips with extraluminal fluid or bubbles were not removed from the test pool, thus all 25 celiac and 25 control videoclips were classified (one for each of 5 locations in each patient).

## Results

**Figure 4:**
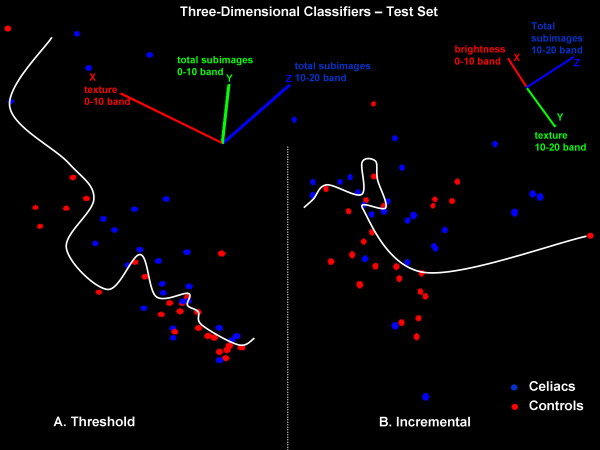
**Classification of a test set of videoclips using A**. Threshold classifier and B. Incremental learning classifier. Nonlinear discriminant functions are shown as white curved lines. The features used for classification and orientation of the coordinate axes for both classifiers are noted at top. Using the same orientation and discriminant functions as for the exemplar set (Figure 3) most celiac (blue) and control (red) videoclip values are correctly classified.

In Figure 4, results of using the classifiers developed from the exemplar set (Figures 2 and 3) are shown for the test set. The axes, projection, and nonlinear discriminant function for the threshold and incremental learning classifiers in Figure 4 correspond to Figure 3. The threshold classifier resulted in a sensitivity of 80% and specificity of 96% for classifying the celiac videoclip scatterpoints (blue) versus controls (red) in the test set (panel A). The incremental classifier resulted in a sensitivity of 88% and specificity of 80% for classifying celiac videoclip scatterpoints versus controls in the test set (panel B). The classifier results for both the exemplar set (see Methods) and for the test set of videoclips are summarized in Table 1. As described in the Methods, the exemplar set is the set of videoclips used to train the classification algorithm (threshold or incremental learning). After use of the exemplar set, the best classifier boundaries were determined (shown in Figures 2 and 3). These same boundaries were then used on a test set of data (Figure 4). Since the sensitivity and specificity remained satisfactory for the test set (80% or more in each case, bottom row in Table 1) it means that the classification boundaries that were developed are useful for distinguishing other celiac versus control videoclips. Hence, it would be expected that complete videoclips of the small intestine in the same patients used in this study, as well as those from other patients, can be classified with similar levels of accuracy.

**Table 1:** Classification Results


**Type**	**Threshold sensitivity**	**Threshold specificity**	**Incremental Sensitivity**	**Incremental specificity**

Exemplar set	91%	95%	100%	90%

Test set	80%	96%	88%	80%

Threshold = threshold classifier, Incremental = incremental classifier

**Figure 5:**
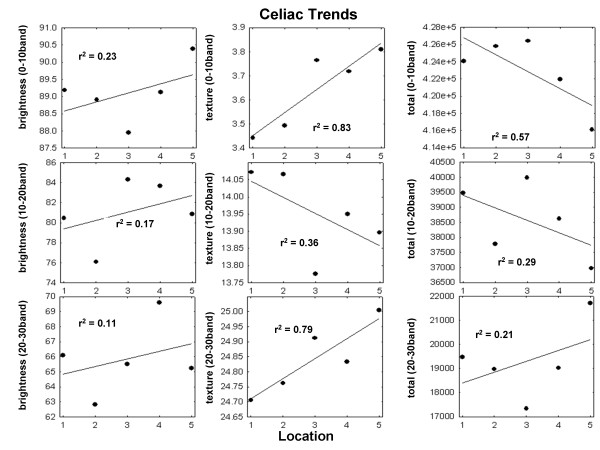
**Mean values of each of the nine features at each small intestinal level were computed from the pooled videoclips of all celiacs, and the resulting scatterplots and linear regression lines are shown**. The change along the small intestine was considered to be a trend for coefficient of determination r^2 ^> 0.50. A trend is therefore defined as an approximately linear change in feature value from location 1 to 5 (i.e. from proximal to distal along the small intestine).

Mean values for each of the nine features at each small intestinal level using all videoclips are shown in Figure 5 (celiacs) and 6 (controls). In each figure, the left column shows mean brightness in each band, the middle column shows mean texture in each band, and the right-hand column shows the total number of subimages in each band. Thus all nine of the original features described in the Methods are graphed. Linear regression lines are drawn in each scatterplot to identify trends from proximal to distal in the small intestine. Any change from location 1 to location 5 was considered to be a trend when the coefficient of determination r^2 ^was greater than 0.50 in these plots. It was found that for the celiac data (Figure 5), significant trends occurred in three of the nine features:

1. increasing texture from location 1 to 5 in the 0-10 band (top middle panel, r^2 ^= 0.83)

2. decreasing total number of subimages from location 1 to 5 in the 0-10 band (top right panel, r^2 ^= 0.57)

3. increasing texture from location 1 to 5 in the 20-30 band (bottom middle panel, r^2 ^= 0.79)

**Figure 6:**
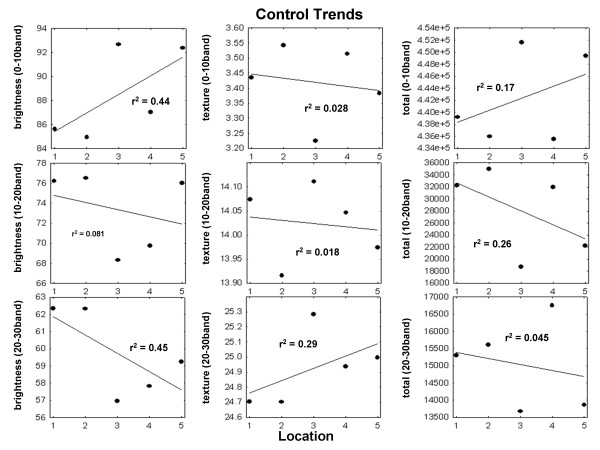
**Mean values of each of the nine features at each small intestinal level were computed from the pooled videoclips of all controls, and the resulting scatterplots and regression lines are shown**. None of these were considered to have significant trends from duodenal bulb to distal ileum (r^2 ^≤ 0.50). The lack of trend in image brightness and texture in the control population suggests a lack of pathologic changes from proximal to distal along the small intestine.

Therefore, trends of increasing texture from proximal to distal along the small intestine occurred in the low and high texture bands for celiac videoclips, while the number of subimages in the low texture band of the celiac videoclips diminished (r^2 ^> 0.5 in each case). No trends occurred in controls - changes occurred from level to level in the small intestine but there was no trend (Figure 6). For celiac videoclips, the mean r^2 ^was 0.40 for all panels (Figure 5) whereas for control videoclips, mean r^2 ^was 0.20 (Figure 6). Therefore, the celiac videocapsule images, but not the controls, tend to have alterations in texture patterns that are linearly related to distance along the small intestine.

## Discussion

In this study, we determined that videocapsule images could be used for identification of celiac disease-associated mucosal abnormalities as compared to mucosal features of individuals without celiac disease, at several small bowel levels. This necessitated determining the time at which the videocapsule passed landmark points, which varied from one patient to the next [6].

### Value of Videocapsule Quantification

The mucosal abnormalities identified in celiacs include decreased number of mucosal folds, mosaic appearance, and scalloping [9]. These features are considered to be highly specific for villous atrophy, thus their presence is indicative of celiac disease. In this study we did not consider the number of mucosal folds, mosaic appearance, and scalloping *per se*. Instead, we measured the brightness and texture of videocapsule images (Figure 1) and found greater variability in celiac patients. The videocapsule camera produces high quality images of the small bowel mucosa (eight-fold magnification) and is able to detect minute mucosal details, including changes in the morphology of the intestinal villi [10]. It is quite likely that changes in the villi, as a consequence of increased degrees of villous atrophy, are being detected by the image analysis-based quantification process. Since the villous atrophy in celiac patients is often very patchy, it would be expected to be manifested as a variable brightness pattern from one location to the next.

In earlier work, videocapsule findings pertaining to the degree of intestinal mucosal atrophy showed only moderate agreement with the histologic pattern, with high sensitivity but a disappointing specificity [11]. In the present study we showed that relatively high sensitivity (mean of 84% for threshold and incremental classifiers, Table 1) and specificity (mean of 88% for threshold and incremental classifiers, Table 1) could be achieved using a test set of videoclips without preprocessing to remove videoclips with air bubbles and extraneous fluids (Figure 4). In another recent study, the overall pooled videocapsule specificity was 98% and sensitivity was 83% [7]. Thus our analysis compares favorably with other recent methods, and it does not require exclusion of any videoclips.

### Additional Applications

Capsule endoscopy is a non-invasive, painless and well-accepted procedure [12]. Thus it can readily be used in patients with known celiac disease for monitoring small bowel healing [12] as well as in patients with alarm symptoms who are otherwise adherent to a gluten-free diet, and in long-term surveillance to detect malignancies [13]. Since real-numbered values are provided by our quantifier, unlike traditional endoscopic methods, it is possible to monitor changes at distal as well as proximal areas of the small bowel (Figures 5 and 6). Additionally, this technique would lend itself to the study of other small intestinal disorders that may present with mucosal atrophy or other visible signs of intestinal damage, for example, inflammatory bowel disease, autoimmune enteritis, tropical sprue, and collagenous sprue.

### Clinical Implications and Future Work

In our study we showed that videocapsule endoscopy can be used to quantitatively assess and classify celiac versus control videoclips. For clinical application, quantitative analysis can be done on the entire video throughout the small intestine to continuously map the entire intestine. Potentially a program could be incorporated into software that is part of the capsule reading device. Thus capsule images could be interpreted by the computer program, vastly expanding the applications of this technology, and, in turn the diagnosis of mucosal diseases such as celiac disease.

Although this study and others suggest that videocapsule endoscopy is an exciting new tool for evaluating the small bowel mucosa in celiac disease, it is typically used as an adjunct to biopsy, to assess the extent of disease and assess for complications of celiac disease. Videocapsule endoscopy is currently the preferred test for imaging the entire length of the small intestine. The use of quantitative techniques may replace biopsy in selected cases especially if there is either an inability, or an unwillingness to undertake routine endoscopy. Videocapsule procedures can be conducted on an outpatient basis [14], and are not believed to interfere with cardiac pacemakers [15]. Moreover, the overall tolerance of videocapsule endoscopy in patients has been impressive, although the capsule must be used with caution in patients with potential obstruction or swallowing disorders [16]. It is also possible to correlate quantitative markers of videocapsule endoscopy such as have been described in this study, with quantitative markers of villous atrophy at the microscopic level [17], the subject of future work. Determining the relationship between the degree of damage throughout the small intestine as measured by quantified parameters, and serum level of antibodies, is also planned for future work.

### Limitations

Videocapsule imaging is a relatively new procedure, thus the number of studies for the assessment of villous atrophy is limited. In our study we pooled the videoclip data from all small intestinal locations - duodenal bulb, distal duodenum, jejunum, ileum, and distal ileum, for classification. In a larger population of celiac and control patients, it should be possible to detect significant differences in videocapsule image data at individual locations. Another possibility to increase accuracy in classification would be to increase the number of features used. It is possible to automate the process of determining which two-dimensional projection of a three-dimensional feature space will yield the most accurate classification with a nonlinear discriminant function.

## Conclusions

Images of the small bowel in celiac patients have spatiotemporal differences in brightness and texture as compared to controls, which can be used in classification and for detecting differences and gradients throughout the small intestine. We determined this by quantifying subimages of each image for a sequence of 200 frames (100s) at each of five locations in celiac and control patients. The analysis of subimages was used to measure spatial variability in each image. The analysis of a sequence of 200 image frames was used to measure temporal variability in small bowel pathology. Sequences were obtained from five small bowel locations, since celiacs can have patchy disease, i.e. variability in the degree of villous atrophy in different regions of the small bowel. Further studies would be helpful to confirm whether our quantitative image analysis methodology can adequately assess global small bowel mucosal alterations and determine whether the extent of small bowel mucosal atrophy correlates with any clinical symptom. Evaluating the reasons for patient refractoriness to gluten-free diet also might benefit from such analysis. Lastly, real-time analysis using the classifiers developed in this study can potentially broaden applicability of videocapsule endoscopy to other etiologies, because of the more objective and quantitative assessment of intestinal injury. Quantitative computer-assisted techniques would remove observer bias and experience as variables in the assessment of mucosal atrophy, and increase the diagnostic value of videocapsule endoscopy in the assessment of celiac disease.

## Competing interests

The authors declare that they have no competing interests.

## Authors' contributions

EJC conceived of and designed the analytical portion of the study and performed the statistical analysis. CAT and SKL designed the clinical portion of the study and obtained the data from celiac and control patients. GB designed the pathology portion of the study and provided pathology data from celiac and control patients. PHRG directed the design and coordination of the study. EJC wrote the draft manuscript and all authors made substantial contributions to its revision. All authors have read and approved the final manuscript.
